# Representation of different skin colors in German nursing textbooks

**DOI:** 10.3205/zma001819

**Published:** 2026-02-17

**Authors:** Viola Marie Nagata, Nils Ohde, Jan Kottner

**Affiliations:** 1 Charité – Universitätsmedizin Berlin, Institute for Clinical Nursing Science, Berlin, Germany

**Keywords:** assessment, education, skin color, nursing

## Abstract

**Background:**

The accurate assessment of skin lesions is one of the core tasks of professional nursing practice. Cutaneous signs depend on the skin color due to the melanin content. Nursing students must therefore learn how skin lesions look in different skin colors.

**Aim:**

The aim of this short report is to describe the current state of representation of different skin colors in German nursing textbooks. The research question was: To what extent are different skin colors represented in German nursing textbooks?

**Methods:**

A systematic analysis of ten German nursing textbooks was conducted. The included images were divided into the ten skin colors according to the Monk Skin Tone Scale. Categories A to D were classified as light, E to G as medium and H to J as dark skin.

**Results:**

Ten textbooks were included and 475 images analyzed. One hundred images (21.1%) showed skin color A, 118 (24.8%) B, 128 (26.9%) C, 94 (19.8%) D, 29 (6.1%) E, five (1.1%) F, one (0.2%) G and none each of H, I and J. In total, 92.6% (n=441) show light skin tones and 7.4% (n=35) medium skin colors.

**Conclusions:**

There is a significant underrepresentation of darker skin colors in images depicting clinical signs and dermatological diseases. This gap in education may lead to delayed symptom recognition, delayed interventions, and possibly wrong diagnoses. Textbooks should be revised to include a broader representation of skin colors, and darker skin colors should be integrated into nursing education to compensate for this limitation.

## Zusammenfassung

**Hintergrund:**

Eine zentrale Aufgabe der professionellen Pflegepraxis ist die präzise Beurteilung von Hautveränderungen. Deren Erscheinungsbild variiert aufgrund des Melaningehalts je nach Hautfarbe. Für Pflegestudierende und -auszubildende ist es daher essenziell zu lernen, wie sich Hautveränderungen auf verschiedenen Hautfarben zeigen.

**Ziel:**

Ziel dieses Kurzbeitrags ist es, den aktuellen Stand der Repräsentation verschiedener Hautfarben in deutschen Pflegelehrbüchern darzustellen. Die Forschungsfrage lautet: In welchem Ausmaß sind verschiedene Hautfarben in deutschen Pflegelehrbüchern repräsentiert?

**Methoden:**

Es wurde eine systematische Analyse von zehn deutschen Pflegelehrbüchern durchgeführt. Die eingeschlossenen Abbildungen wurden gemäß der Monk-Hautfarben-Skala in zehn Hautfarben unterteilt. Die Kategorien A bis D wurden als hell, E bis G als mittel und H bis J als dunkel eingestuft.

**Ergebnisse:**

Es wurden zehn Lehrbücher einbezogen und 475 Bilder analysiert. Einhundert Bilder (21,1%) zeigten Hautfarbe A, 118 (24,8%) B, 128 (26,9%) C, 94 (19,8%) D, 29 (6,1%) E, fünf (1,1%) F, eins (0,2%) G und keine H, I und J. Insgesamt zeigen 92,6% (n=441) helle Hauttöne und 7,4% (n=35) mittlere Hautfarben.

**Schlussfolgerungen:**

In Abbildungen, die klinische Symptome und Hautkrankheiten darstellen, sind dunklere Hautfarben deutlich unterrepräsentiert. Diese Lücke in der Ausbildung kann zu einer verzögerten Identifikation von Symptomen, verspäteten Einleitung von Interventionen und möglicherweise zu Fehldiagnosen führen. Lehrbücher sollten überarbeitet werden, um eine breitere Darstellung von Hautfarben zu ermöglichen, und dunklere Hautfarben sollten in die Pflegeausbildung integriert werden, um diese Einschränkung auszugleichen.

## Background

The systematic assessment of patients and care receivers is a core responsibility of professional nursing. This includes the assessment of skin conditions. Clinical signs such as scaling, papules, pustules, erosions, excoriations, or erythema may indicate dry skin, inflammation or the development of pressure ulcers [1], [2]. Education is important to enhance skin assessment skills. The detection and assessment of cutaneous signs depend, among other factors, on the pigmentation of the epidermis and can be particularly challenging in individuals with higher melanin levels [3], [4], [5]. Therefore, having knowledge about the different possible manifestations of dermatoses in skin of color is essential for clinical nursing assessments [6].

Textbooks are used by nursing apprentices, students, and nurse educators, and serve as foundational resources for education. They offer the opportunity to expose future nurses to the variety of different possible manifestations of dermatoses in skin of color, thereby fostering skills for the appropriate treatment of patients regardless of skin color [7]. However, studies from the UK and the USA have shown a significant underrepresentation of darker skin tones in journals and textbooks [8], [9], [10].

Research on the representation of skin tones in textbooks and journals is new and focused on medical literature so far [10]. To date, only one American study examined nursing textbooks [11]. This study is the first to explore the extent to which German nursing textbooks are suitable for teaching symptom recognition across different skin tones (see table 1 (Tab. 1)).

## Method

To examine how skin tones are represented in German nursing education, a study was conducted analyzing the extent to which different skin tones appear in images depicting cutaneous symptoms in German nursing textbooks. Ten textbooks, aimed at nursing apprentices and students, were selected for the analysis (see table 1 (Tab. 1)). To accurately reflect the current state of representation, all textbooks were analyzed in their most recent editions.

**Figure 1:**
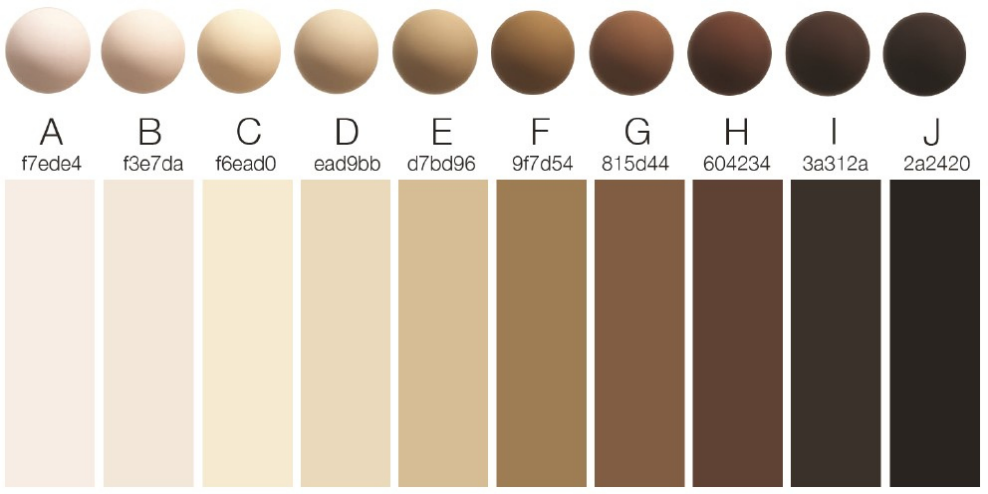
Monk skin tone scale [16]

A list of keywords was used to identify relevant chapters and illustrations. This list included key dermatological symptoms [12], common and specific dermatological diseases [13], [14], as well as cutaneous manifestations of internal diseases [15]. Additionally, symptoms that are particularly relevant due to their variable presentation on darker skin – such as cyanosis, pressure injuries, inflammation, erythema, and jaundice – were included [7]. The skin tones depicted in the selected images were classified using the Monk Skin Tone (MST) scale (see figure 1 (Fig. 1)). This classification tool represents a broad spectrum of skin tones [16], consisting of ten categories: four light (A to D), three medium (E to G) and three dark skin tones (H to J) [17].

## Results

A total of 475 images from the ten nursing textbooks were analyzed and categorized according to the MST scale (see table 2 (Tab. 2)). Furthermore, the images were assigned to 48 categories of clinical signs and dermatological diseases (see table 3 (Tab. 3)). The results reveal that light skin tones are predominant, a trend observed across all textbooks. Overall, light skin tones accounted for 92.6% (n=440) of all images. Dark skin tones (H, I and J) were not represented in any of the textbooks, including those with the highest number of images. This points to a significant gap in the visual representation of dark skin. Medium skin tones were marginally represented, accounting for 7.4% (n=35) of images. Particularly striking is the fact that of the 48 categories of clinical signs and dermatological diseases, 34 were depicted exclusively on light skin.

## Discussion

The lack of images depicting dark skin may limit the competence of nursing apprentices and students to recognize cutaneous lesions in darker skin. This gap in education may lead to delayed symptom recognition, delayed interventions, and possibly wrong diagnoses. When clinical signs such as cyanosis, allergic reaction, or erythema associated with a stage 1 pressure ulcer are not promptly recognized, delayed diagnosis may affect patient outcomes.

The ICN Code of Ethics emphasizes that nurses, through their professional practice, must advocate for equal treatment and equity in both healthcare and society, thereby upholding their professional values [18]. This responsibility is particularly important because nurses play a vital role in assessing skin conditions [2], [19].

Nursing educators have a responsibility to address deficits within healthcare systems. To help close existing gaps, educators should actively raise awareness of how symptoms may present differently on various skin tones. Comparative imagery featuring darker skin tones could support this effort by visualizing these variations, promoting their normalization, and training future nurses in diversity-sensitive symptom recognition [4]. Introducing this awareness early in education may support person-centered care.

However, it remains unclear whether and to what extent nurse educators are currently addressing this issue, as no studies have yet investigated this topic.

This study included only ten nursing textbooks and therefore may not be fully representative of all textbooks currently used in German nursing education. Furthermore, due to the focus on skin-related symptoms, not all images within the textbooks were included in the analysis.

The analysis of the selected nursing textbooks reveals a significant underrepresentation of darker skin tones in images depicting clinical signs and dermatological diseases. The predominance of light skin tones suggests that nursing students may not be adequately prepared to provide diversity-sensitive care. Given that nurses routinely care for patients with a wide range of skin tones, this shortcoming poses a risk of suboptimal care. A lack of visual references may lead to uncertainty in clinical practice or even result in misdiagnoses.

Previous research has yielded comparative findings, with the representation of dark skin tones constituting the primary difference from the current study. While 12.3% of images in American nursing textbooks [11], 1.1% to 2.8% in American medical textbooks [20], [21], and 0.02% in German dermatological textbooks [22] depict dark skin tones, there is an absolute lack of dark skin tones in the analyzed German nursing textbooks.

To address this issue, textbooks should be revised to include a broader representation of skin tones, and further research is needed to examine how different skin tones are integrated into nursing education. Diversity-sensitive training is essential to ensure safe, equitable, and appropriate care for all patients and care recipients, regardless of skin color. These findings underscore the need for immediate curricular reforms to ensure equitable clinical competence in future nursing professionals.

## Authors’ ORCIDs

- Nils Ohde: [0009-0001-5200-1019]
- Jan Kottner: [0000-0003-0750-3818]

## Competing interests

The authors declare that they have no competing interests. 

## Figures and Tables

**Table 1:**
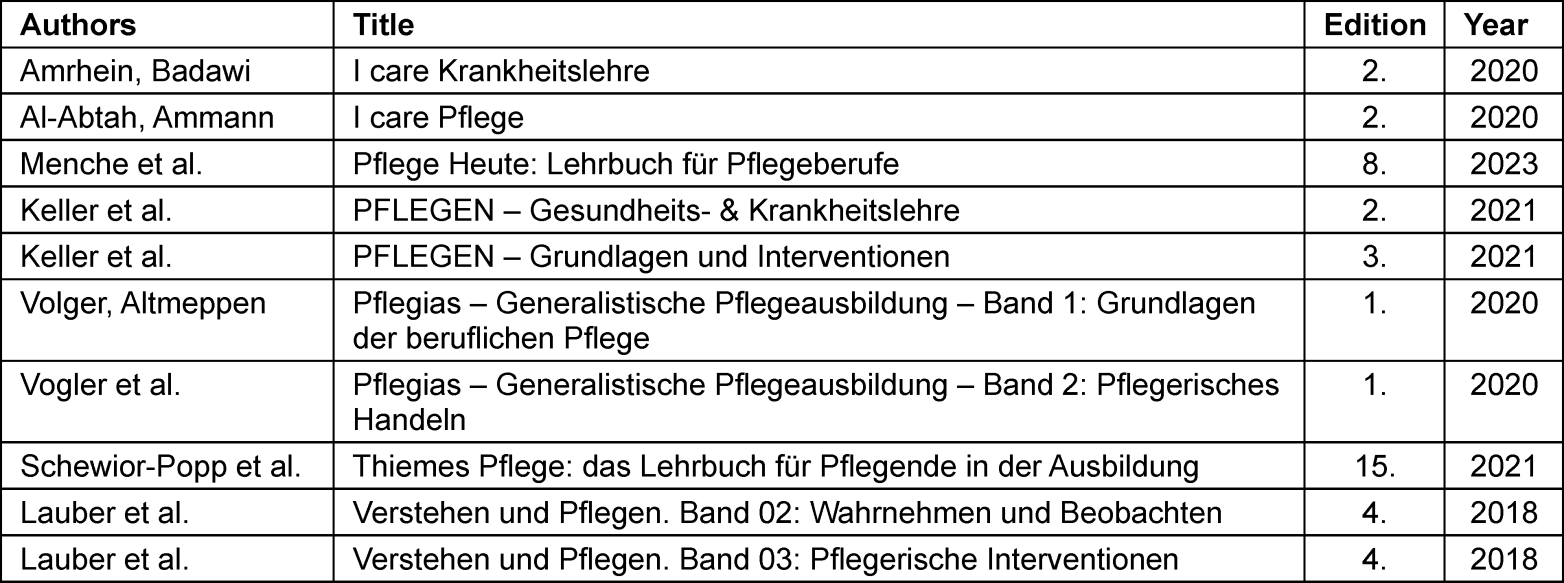
Selected nursing textbooks

**Table 2:**
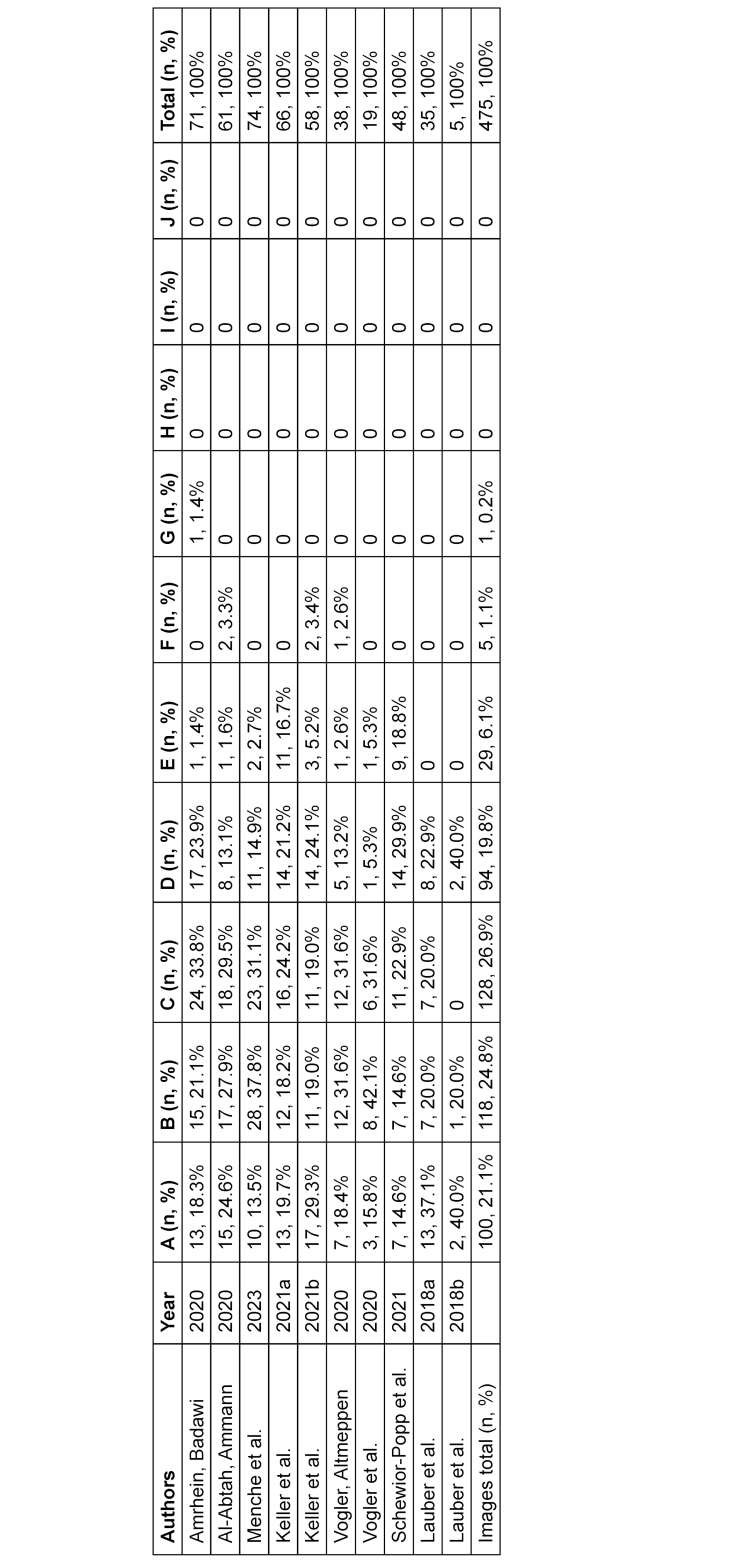
Skin colors per textbook (n, %)

**Table 3:**
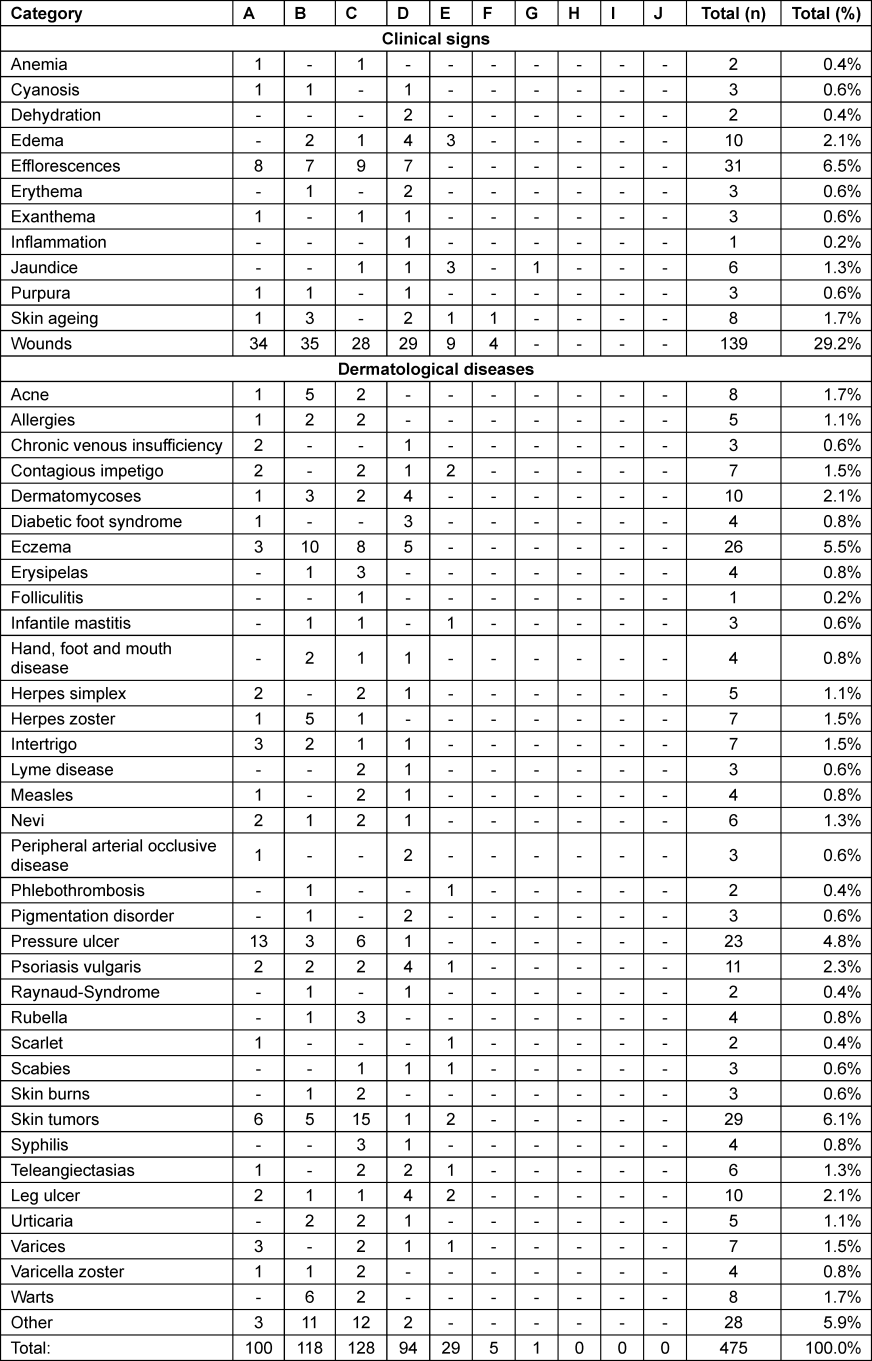
Skin colors per category
